# A phase I trial of ganetespib in combination with paclitaxel and trastuzumab in patients with human epidermal growth factor receptor-2 (HER2)-positive metastatic breast cancer

**DOI:** 10.1186/s13058-017-0879-5

**Published:** 2017-08-02

**Authors:** Komal Jhaveri, Rui Wang, Eleonora Teplinsky, Sarat Chandarlapaty, David Solit, Karen Cadoo, James Speyer, Gabriella D’Andrea, Sylvia Adams, Sujata Patil, Sofia Haque, Tara O’Neill, Kent Friedman, Francisco J. Esteva, Clifford Hudis, Shanu Modi

**Affiliations:** 1 Memorial Sloan-Kettering Cancer Center, New York, NY USA; 2 Valley–Mount Sinai Comprehensive Cancer Care, Paramus, NJ USA; 3 Laura and Isaac Perlmutter Cancer Center at NYU Langone Medical Center, New York, NY USA

**Keywords:** Ganetespib, Paclitaxel, Trastuzumab, HSP90 inhibitor, HER2, Metastatic breast cancer, Phase I trial

## Abstract

**Background:**

Targeted therapies in HER2-positive metastatic breast cancer significantly improve outcomes but efficacy is limited by therapeutic resistance. HER2 is an acutely sensitive Heat Shock Protein 90 (HSP90) client and HSP90 inhibition can overcome trastuzumab resistance. Preclinical data suggest that HSP90 inhibition is synergistic with taxanes with the potential for significant clinical activity. We therefore tested ganetespib, a HSP90 inhibitor, in combination with paclitaxel and trastuzumab in patients with trastuzumab-refractory HER2-positive metastatic breast cancer.

**Methods:**

In this phase I dose-escalation study, patients with trastuzumab-resistant HER2-positive metastatic breast cancer received weekly trastuzumab (2 mg/kg) and paclitaxel (80 mg/m^2^) on days 1, 8, 15, and 22 of a 28-day cycle with escalating doses of ganetespib (100 mg/m^2^, 150 mg/m^2^, and a third cohort of 125 mg/m^2^ if needed) on days 1, 8, and 15. Therapy was continued until disease progression or toxicity. The primary objective was to establish the safety and maximum tolerated dose and/or recommended phase II dose (RP2D) of this therapy. The secondary objectives included evaluation of the effects of ganetespib on the pharmacokinetics of paclitaxel, and to make a preliminary assessment of the efficacy of the combination therapy.

**Results:**

Dose escalation was completed for the two main cohorts without any observed dose-limiting toxicities. Nine patients received treatment. The median prior lines of anti-HER2 therapy numbered three (range 2–4), including prior pertuzumab in 9/9 patients and ado-trastuzumab emtansine (T-DM1) in 8/9 patients. The most common grade 1/2 adverse events (AEs) were diarrhea, fatigue, anemia, and rash. There were no grade 4 AEs related to ganetespib. The overall response rate was 22% (2/9 patients had partial response) and stable disease was seen in 56% (5/9 patients). The clinical benefit rate was 44% (4/9 patients). The median progression-free survival was 20 weeks (range 8–55).

**Conclusion:**

The RP2D of ganetespib is 150 mg/m^2^ in combination with weekly paclitaxel plus trastuzumab. The combination was safe and well tolerated. Despite prior taxanes, pertuzumab, and T-DM1, clinical activity of this triplet regimen in this heavily pretreated cohort is promising and warrants further study in HER2-positive metastatic breast cancer.

**Trial registration:**

ClinicalTrials.gov NCT02060253. Registered 30 January 2014.

## Background

HER2-positive disease accounts for 15–20% of breast cancers, and traditionally has an aggressive clinical course and inferior survival outcome [1–3]. Clinical benefits from trastuzumab and other anti-HER2 therapies have greatly improved results for patients with HER2-positive disease, but are limited by the development of resistance [4].

HSP90 belongs to a class of molecular chaperone proteins that help modulate cellular responses to environmental stress [5]. In particular, HSP90 regulates the folding, stability, and function of many cellular proteins including several receptor tyrosine kinases (RTKS). Inhibition of HSP90 is believed to cause these client proteins to adopt conformations which stimulate their ubiquitination and degradation by the proteasome [6–10]. HER2 is one of the most sensitive HSP90 clients, and HER2-amplified breast cancer cells are potently inhibited by geldanamycin, the prototype HSP90 inhibitor [11, 12]. Multiple first-generation geldanamycin-derived HSP90 inhibitors have been evaluated for the treatment of HER2-positive breast cancer [13–16]. The greatest clinical activity was reported with tanespimycin (17-AAG) in combination with trastuzumab in trastuzumab-refractory HER2-positive metastatic breast cancer with a response rate of 22% and a clinical benefit rate (CBR) of 59% [15, 16]. Another phase I trial of alvespimycin plus trastuzumab reported one partial response (PR) and six cases of stable disease (SD) lasting >6 months in patients with HER2-positive metastatic breast cancer [14]. A phase II study of retaspimycin (IPI-504) in combination with trastuzumab was also found to be well tolerated with modest anti-tumor effects (62% of patients had stable disease) [13]. Ganetespib ((5-(2,4-dihydroxy-5-(1-methylethyl)phenyl)-4-(1-methyl-1H-indol-5-yl)-2,4-dihydro-(1,2,4)triazol-3-one)) is a second-generation synthetic small molecule that binds to the ATP pocket in the N-terminus of HSP90 [17–19], is structurally unrelated to geldanamycin-derived inhibitors, and has demonstrated significant activity for downregulating HSP90 client protein levels preclinically. Specifically, ganetespib showed stronger anti-tumor activity compared to tanespimycin over a broader range of breast cancer subtypes, including HER2-normal cancer and triple-negative breast cancer (TNBC), with a more favorable safety profile, including lack of hepatotoxicity and ocular toxicity [18, 19]. We recently reported a single-arm phase II study of single-agent ganetespib in unselected patients with heavily treated metastatic breast cancer who received up to three lines of chemotherapy [19]. That study did not meet the prespecified criteria for overall response in the first stage in a heavily pretreated group of patients; however, there were two confirmed PRs and six cases of SD in patients with HER2-positive, trastuzumab-refractory metastatic breast cancer that further justified its study for this subtype of breast cancer [19].

A novel approach to the treatment of metastatic breast cancer is the combination of HSP90 inhibitors and taxanes. Taxanes disrupt an essential structural component (microtubules) of mitosis, and HSP90 inhibitors impact the regulatory (checkpoint) proteins controlling progression through the cell cycle [20]. In addition, both drugs disrupt other critical facets of cell growth and proliferation, adding to their potential combined efficacy [20–22]. When paclitaxel was given with HSP90 inhibitors in nude mice bearing tumor xenografts, there was a 5-fold–22-fold enhancement of cytotoxicity [20]. Maximal synergistic anti-tumor activity was seen in breast cancer xenografts when tanespimycin and paclitaxel were administered sequentially on the same day [21]. Importantly, the addition of tanespimycin to cells after exposure to paclitaxel significantly increased both the activation of caspases 9 and 3 and thus apoptosis, indicating that the sequence of drugs (paclitaxel followed by HSP90 inhibitor) matters and influences efficacy [22].

The primary objective of this study was to establish the safety, tolerability, maximum tolerated dose (MTD), and/or recommended phase II dose (RP2D) of ganetespib plus paclitaxel in conjunction with trastuzumab in patients with HER2-positive metastatic breast cancer. The secondary objectives included evaluation of the possible effects of ganetespib on the pharmacokinetics (PK) of paclitaxel, and to make a preliminary assessment of the efficacy of the combination.

## Methods

### Study design and patient selection

Patients were eligible if they were aged ≥ 18 years, had locally advanced or metastatic HER2-positive disease (defined as FISH ratio ≥ 2.0 or immunohistochemistry (IHC) 3+), ECOG ≤ 2, measurable disease per RECIST 1.1 [23], and adequate end organ function (defined as hemoglobin ≥ 9 g/dl, absolute neutrophil count (ANC) ≥ 1.5 × 10^9^/L, platelets ≥ 100 × 10^9^/L, bilirubin ≤ 1.5 upper limit of normal (ULN), aspartate aminotransferase (AST) and alanine aminotransferase (ALT) ≤ 2.5 ULN, and serum creatinine ≤ 1.5 ULN). Patients must have received prior trastuzumab and those with estrogen receptor (ER)-positive disease must have received prior endocrine therapy. Any number of prior lines of chemotherapy in the metastatic setting was allowed. Progression on prior treatment with pertuzumab and T-DM1 was required (unless heavily pretreated prior to FDA approval of pertuzumab for first-line treatment of HER2-positive metastatic breast cancer (6/2012) and/or T-DM1 (2/2013)).

Patients were excluded if they were pregnant or lactating, had prior grade 3 hypersensitivity to cremophor or trastuzumab, had prior HSP90 inhibitor therapy, had active central nervous system metastases, New York Heart Association (NYHA) class III/IV congestive heart failure requiring active treatment, left ventricular ejection fraction (LVEF) < 50% at baseline, baseline QTc > 470 milliseconds, or grade ≥ 2 peripheral neuropathy, were on any medications known to prolong QTc, had preexisting left bundle branch block (LBBB), history of uncontrolled dysrhythmias, or a requirement for antiarrhythmics, had myocardial infarction (MI) or ischemic heart disease within 6 months, or had known active infection with HIV or hepatitis B or C viruses.

The study was approved by the institutional research ethics board of Memorial Sloan Kettering Cancer Center and New York University Langone Medical Center. All participants gave informed consent before they entered the study.

### Study treatment

Patients received intravenous infusions of trastuzumab and paclitaxel with ganetespib on days 1, 8, and 15 and of trastuzumab and paclitaxel on day 22 of a 28-day cycle. The sequence of administration was trastuzumab (2 mg/kg) followed by paclitaxel (80 mg/m^2^) followed by ganetespib. If the patient’s last dose of trastuzumab was >21 days before enrollment, they received a loading dose of trastuzumab at 4 mg/kg over 90 minutes. Ganetespib was then administered intravenously over 60 minutes. The starting dose of ganetespib was 100 mg/m^2^, and if there were no DLTs the next cohort would escalate to 150 mg/m^2^. A further dose level of 125 mg/m^2^ was incorporated in the circumstance of good tolerance of the 100 mg/m^2^ but poor tolerance of the 150 mg/m^2^ dose levels. There was no dose escalation for paclitaxel and trastuzumab. Therapy was continued until disease progression or unacceptable toxicity.

### Toxicity assessment and dose reductions

Patients were examined and assessed for toxicities during and prior to each cycle. Toxicity was graded according to National Cancer Institute (NCI) CTCAE version 4.0 (http://ctep.cancer.gov/protocolDevelopment/electronic_applications/ctc.htm#ctc_40). Patients were evaluated for DLT during cycle 1. DLT was defined as any drug-related grade ≥ 4 nonhematologic adverse events (AEs) or any grade 3 nonhematologic AEs not improving to baseline or grade ≤ 1 by day 14; grade 4 neutropenia lasting ≥ 7 days, or febrile neutropenia, grade 4 thrombocytopenia, or any grade 3 thrombocytopenia that has not recovered to grade ≤ 2 by day 7; or any treatment-related toxicity prompting a dose reduction of ganetespib during the DLT observation period.

Paclitaxel dose reductions were not permitted during the DLT observation period. For dosing beyond cycle 1, paclitaxel was held if patients experienced any other grade 3 or 4 toxicity thought to be related to paclitaxel until symptoms resolved to grade 1/baseline grade. One dose reduction for paclitaxel to 65 mg/m^2^ was permitted.

### Assessment of treatment response

Patients were evaluated for response initially after two cycles and then every three cycles thereafter using the RECIST criteria [23]. All patients with PR or complete response (CR) were required to have confirmation of response 4 weeks after the criteria for response were first met. The best overall response was defined as the best response recorded from the start of treatment until disease progression or withdrawal from the study. All patients who received at least one full cycle (4 weeks) of ganetespib, paclitaxel, and trastuzumab and had a follow-up assessment were evaluable for response. The CBR was defined as the proportion of patients whose best overall response, according to RECIST, was CR, PR, or SD lasting for at least 24 weeks.

### Pharmacokinetics of paclitaxel assessment

Blood samples for determination of plasma concentrations of paclitaxel were collected on cycle 1, day 8 through cycle 1, day 9 at the following time points after trastuzumab infusion and relative to the start of the paclitaxel infusion: 5 minutes prior to treatment, and 30 and 60 minutes (immediately prior to stopping the paclitaxel infusion pump and starting the ganetespib infusion) after start of paclitaxel infusion. Blood samples were also collected at the following time points after starting ganetespib infusion: 1.5, 2, 4, 7, 21, 24, 27, and 31 hours. Each blood sample (3–5 ml) was collected in sodium heparin tubes and transferred into two polypropylene tubes (1 ml each) and stored at –80 °C. PK were performed in collaboration with Synta Pharmaceuticals, Inc. PK parameters of paclitaxel (such as the area under the curve (AUC) and the maximum serum concentration (Cmax)) were examined descriptively to evaluate the effect of ganetespib on these measures.

## Results

### Patients and treatment

Nine patients were enrolled into two dose cohorts: three patients at 100 mg/m^2^ and six patients at 150 mg/m^2^. Patient demographics are presented in Table 1. The median age was 46 (range 29–65) years and median ECOG performance status was 0 (range 0–1, Table 1). Seven out of nine patients had ER-positive/HER2-positive breast cancer, and two patients had ER-negative/HER2-positive breast cancer. Patients were heavily pretreated with a median of three (range 2–6) lines of chemotherapy and three prior (range 2–4) anti-HER2 therapies in the metastatic setting, including prior pertuzumab in 9/9 patients and T-DM1 in 8/9 patients. For patients with ER-positive breast cancer, the median number of prior lines of endocrine therapy in the metastatic setting was one (range 1–3, Table 1).

**Table 1:** Patient demographics

Baseline characteristic	Number
Total enrolled	9
Median (range) age (years) at study enrollment	46 (29–65)
Subtype	
ER+/HER2+	7
ER–/HER2+	2
Median ECOG performance status	0 (0–1)
Prior lines of chemotherapy in the metastatic setting, median (range)	3 (2–6)
Prior lines of endocrine therapies in the metastatic setting, median (range)	1 (1–3)
Prior number of anti-HER2 agents in the metastatic setting, median (range)^a^	3 (2–4)

*–* negative, *+* positive, *ECOG* Eastern Cooperative Oncology Group, *ER* estrogen receptor, *HER2* human epidermal growth factor receptor 2, *T-DM1* ado-trastuzumab emtansine

^a^Including prior pertuzumab in 9/9 patients and T-DM1 in 8/9 patients

### Overall safety

**Fig. 1:**
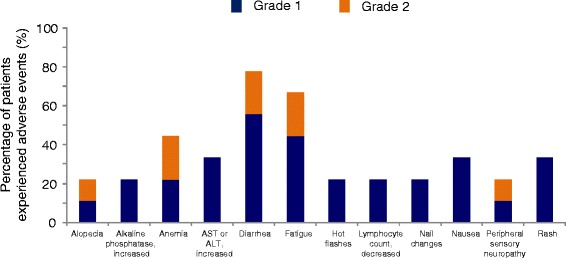
Most common ganetespib-related grade 1/2 AEs in ≥20% of patients. None of the nine patients experienced DLTs. *ALT* alanine aminotransferase, *AST* aspartate transaminase

All nine patients were included in the safety analysis. No patients voluntarily withdrew from the study and no patients were taken off study due to toxicities. There were no grade 4 AEs related to ganetespib. The most common drug-related AEs were diarrhea (grade 1/2, 78%), fatigue (grade 1/2, 67%), anemia (grade 1/2, 44%), rash (grade 1/2, 32%), elevated AST or ALT (grade 1/2, 32%), and nausea (grade 1/2, 32%) (Fig. 1). The diarrhea was well managed with pre/supportive medications (Fig. 1). Ganetespib-related grade 3 AEs were minimal, and included pruritus (one patient), decreased phosphorus (one patient), and increased ALT (one patient), all of which resolved with dose delays of up to 1 week (Table 2). One patient had grade 3 blurred vision and dry eyes which was attributable to paclitaxel; she had experienced these symptoms with prior paclitaxel therapy and they resolved with the discontinuation of paclitaxel. There were no further episodes of blurry vision when the paclitaxel dose was reduced to 65 mg/m^2^ in this patient. No patients on study required ganetespib dose reductions. There were no deaths on study.

**Table 2:** Ganetespib-related grade 3 adverse events

*N*	Ganetespib-related grade 3 adverse events	Outcome
1^a^	Grade 3 blurred vision/grade 3 dry eyes (in same patient)^a^	Blurred vision: dose held for 1 week until ≤ grade 1 and paclitaxel dose reduced to 65 mg/m^2^/ Dry eyes: no action taken: resolved in 1 week
1	Grade 3 pruritus	While attributed to ganetespib, symptoms resolved after paclitaxel was held
1	Grade 3 phosphorus, decreased	No action taken; resolved in 1 day
1	Grade 3 ALT, increased	Ganetespib and paclitaxel dose held for 1 week; resolved in 1 week

*ALT* alanine aminotransferase

^a^One patient had blurred vision and dry eyes which were felt to be attributable to paclitaxel based on her having similar symptoms when treated with paclitaxel in the past. She was followed by an ophthalmologist for this toxicity which resolved with the discontinuation of paclitaxel

### Anti-tumor activity

**Fig. 2:**
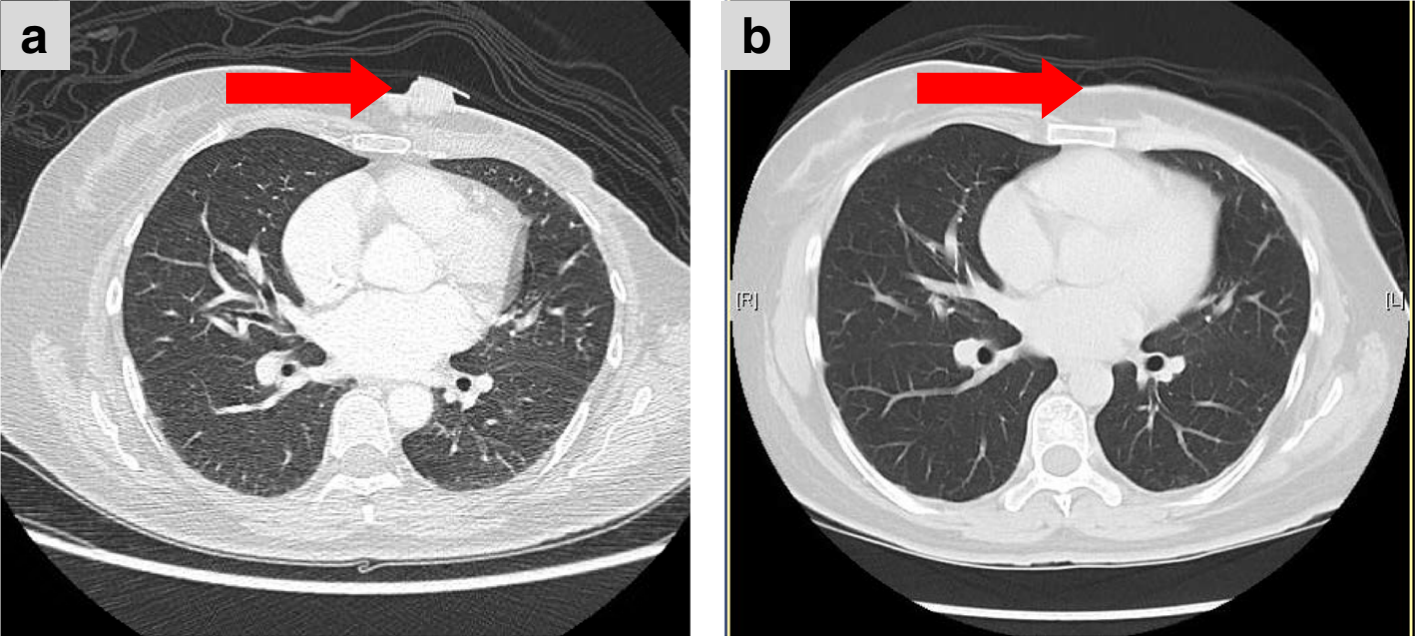
Baseline and follow-up CT scans. Baseline (**a**) and follow-up (**b**) CT scans of a 43-year-old patient with left chest wall soft tissue metastases

Of the nine patients enrolled, confirmed partial tumor responses were achieved in two of the nine patients, both in the 150 mg/m^2^ cohort for an overall response rate (ORR) of 22%. Five additional patients achieved SD (56%), and duration of SD ranged from 11 to 29 weeks. The CBR was 44% (4/9 patients). Representative CT scans prior to and after this triplet regimen in a patient with chest wall soft tissue metastasis who achieved PR are shown in Fig. 2.

### Pharmacokinetics

**Fig. 3:**
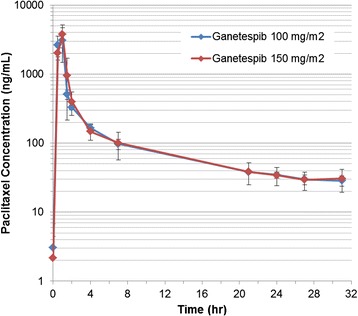
Paclitaxel PK. Preliminary paclitaxel PK data for the nine study patients are not appreciably different from those reported in the literature

PK evaluations were carried out in all nine patients to evaluate the effect of ganetespib on the paclitaxel absorption. Table 3 presents the PK parameters for ganetespib at cycle 1, day 8 through cycle 1, day 9. Trastuzumab was administered before the PK samples were taken. Paclitaxel PK data are not appreciably different from those reported in the literature taking into account differences in dose and sampling scheme (Table 3, Fig. 3) [24, 25]. There was no effect of ganetespib on paclitaxel PK at the dose of 150 mg/m^2^. For paclitaxel, the AUC was 6280 h*ng/ml, the elimination half-life (T_1/2_) was 13.6 hours, the Cmax was 3750 ng/ml, the clearance was 13.4 L/h/m^2^, and the mean resident time (MRT) was 9.0 hours (Table 3).

**Table 3:** Paclitaxel pharmacokinetics

Ganetespib (mg/m^2^)	Number	Cmax	Tmax	T_1/2_	AUC	CL	MRT
		(ng/ml)	(hours)	(hours)	(h*ng/ml)	(L/h/m^2^)	(hours)
100	3	3340 ± 1270	0.8 ± 0.3	12.9 ± 0.4	5900 ± 1440	14.2 ± 3.5	8.5 ± 0.8
150	6	3750 ± 1370	1.0 ± 0.0	13.6 ± 3.3	6280 ± 1430	13.4 ± 3.2	9.0 ± 4.0

*Cmax* maximum serum concentration, *Tmax* time of maximum serum concentration observed, *T*_*1*/2_ half-life *AUC* area under the curve, *CL* clearance, *MRT* mean resident time

## Discussion

HSP90 is a molecular chaperone, supporting a number of cellular onco-proteins that are critical for cancer cell survival and progression. Inhibition of HSP90 therefore has the potential to simultaneously disrupt multiple signaling pathways in cancer cells and hence has been an extensively investigated and highly sought-after strategy for cancer therapy [5–10, 26]. Previous studies have shown clinical anti-tumor activity with various different HSP90 inhibitors [13–19]. Preclinical studies have shown synergistic anti-tumor effects with no additional adverse effects when HSP90 inhibitors have been combined with taxanes [18–20]. The randomized phase II GALAXY-I trial of ganetespib and docetaxel demonstrated improved overall survival in the combination arm compared with docetaxel alone, for the second-line setting in patients with advanced NSCLC who were at least 6 months from initial diagnosis of advanced disease [27].

This phase Ib trial is the first to report on the use of ganetespib in combination with paclitaxel and trastuzumab for patients with HER2-positive metastatic breast cancer. Consistent with the preclinical experience, the combination of paclitaxel and trastuzumab with ganetespib was well tolerated. The AEs observed were largely grade 1 or 2 in nature and included diarrhea, fatigue, anemia, rash, and nausea. This study and our previously reported phase II study with single-agent ganetespib [19] together did not reveal significant off-target DLTs such as hepatotoxicity and cardiotoxicity (congestive heart failure, QTc changes). Furthermore, compared to other HSP90 inhibitors, we observed a low rate of ocular toxicity/retinal injury in this trial. There was one patient who experienced grade 3 dry eye and blurry vision, but her symptoms were related to paclitaxel and resolved with its discontinuation.

The combination of paclitaxel, trastuzumab, and ganetespib was clinically active, with two PRs (22%) in patients with heavily pretreated trastuzumab-refractory HER2-positive metastatic breast cancer. Additionally, five patients achieved SD (56%) with a CBR of 44% (4/9 patients). Notably, in our previous phase II single-agent ganetespib trial, the ORR rate of ganetespib was 15% with two PRs in trastuzumab-refractory ER+/HER2+ metastatic breast cancer. There were seven SDs of which six were seen in patients with HER2-positive metastatic breast cancer and one in a patient with TNBC.

Aside from ganetespib, there are a number of other second-generation HSP90 inhibitors that are in preclinical or clinical testing, including resorcinol derivatives (NVP-AUY922, AT-13387, KW-2478), purine derivatives (CNF2024/BIIB021, PU-H71, MPC-3100, CUDC-305), and other inhibitors including SNX-5422, NVP-HSP990, and XL888 [28–42]. Some of these agents have been tested in patients with breast cancer. For example, a phase II expansion trial of single-agent NVP-AUY922 given intravenously to patients with HER2-positive and ER-positive breast cancer reported two partial metabolic responses on FDG-PET and one confirmed PR by RECIST among the 10 patients enrolled [29]. PU-H71, a purine derivative that is thought to be active in TNBC, has been studied in a phase I trial of patients with advanced solid tumors and lymphoma. The trial was completed recently and revealed a favorable safety profile and evidence of anti-tumor activity across a broad range of tumor types [43]. A phase Ib study of the combination of PU-H71 and nab-paclitaxel is planned for patients with HER2-negative metastatic breast cancer, including patients with triple-negative disease at Memorial Sloan Kettering Cancer Center.

Another heavily studied area in the field of HSP90 inhibitors is the identification of biomarkers and companion diagnostic assays which are crucial to identify patients most likely to respond to therapy. Our group conducted a retrospective study to explore potential biomarkers in patients treated with HSP90 inhibitors. Among many potential candidates analyzed (HER2, HSP90, HSP70, phosphotension homolog), HER2 was found to be the most important individual biomarker and the only one with correlation to response with HSP90 inhibitor therapy [44]. While tumor biopsies have been undertaken in some trials and can serve as a useful tool to establish target modulation, they provide only static information for a small part of the tumor and cannot account for the heterogeneity of metastatic tumor burden. In contrast, molecular imaging biomarkers allow for serial noninvasive assessments including providing data regarding spatial and temporal tumor uptake and retention. Additionally, they have the potential to serve as a predictive biomarker of response. In fact, direct molecular imaging using the labeled drug itself can guide patient selection, help measure tumor PK, and optimize the dose and schedule for this class of agents. For instance, a unique feature of PU-H71 is that it has an endogenous iodine atom (^127^I), which was replaced with the PET radionuclide ^124^I to result in the imaging agent, ^124^I-PU-H71 [45]. Importantly, the PET agent is molecularly identical to PU-H71 and its half-life of 4.02 days makes serial imaging practical. A phase 0, first-in-human trial of ^124^I-PU-H71 in patients with advanced solid tumors and lymphoma not only determined the microdose biodistribution of PU-H71 but also ensured tracer avidity of tumors [45]. Tracer uptake at the metastatic tumor sites in this study correlated well with baseline CT and/or FDG-PET scans. The phase I clinical trial of PU-H71 in patients with advanced solid tumors and lymphoma also incorporated ^124^I-PU-H71 PET to determine tumor PK and the intratumoral drug concentration. Findings from this study showed close concordance between intratumoral drug concentrations as determined by tumor biopsies with estimated measurements from ^124^I-PU-H71 PET [46], highlighting the potential role of ^124^I-PU-H71 PET as a biomarker to visualize PU-H71 uptake and to estimate intratumoral concentrations of the inhibitor.

## Conclusion

The combination of ganetespib with paclitaxel plus trastuzumab is well tolerated, safe, and active with a RP2D of ganetespib at 150 mg/m^2^ in this triplet therapy. Based on the clinical activity in this heavily pretreated population, this combination warrants further study in HER2-positive metastatic breast cancer. In addition, further exploration of biomarkers that predict sensitivity to ganetespib is a critical step for the optimal clinical development of this and other HSP90 inhibitors.

## Abbreviations

AE: Adverse event
ALT: Alanine aminotransferase
ANC: Absolute neutrophil count
AST: Aspartate aminotransferase
AUC: Area under the curve
CBR: Clinical benefit rate
Cmax: Maximum serum concentration
CR: complete response
CTCAE: Common terminology criteria for adverse events
DLT: Dose-limiting toxicity
ECOG: Eastern Cooperative Group
ER: Estrogen receptor
FDA: Food and Drug Administration
FDG-PET: Fluorodeoxyglucose-positron emission tomography
FISH: Fluorescence in-situ hybridization
HER2: Human epidermal growth factor receptor 2
HSP90: Heat Shock Protein 90
IHC: Immunohistochemistry
LBBB: Left bundle branch block
LVEF: Left ventricular ejection fraction
MRT: Mean resident time
MTD: Maximum tolerated dose
NCI: National Cancer Institute
NSCLC: Nonsmall cell lung cancer
NYHA: New York Heart Association
ORR: Overall response rate
PK: Pharmacokinetic
PR: Partial response
RECIST: Response evaluation criteria in solid tumors
RP2D: Recommended phase II dose
RTK: Receptor tyrosine kinase
SD: Stable disease
T_1/2_: Half-life
T-DM1: Ado-trastuzumab emtansine
TNBC: Triple-negative breast cancer
ULN: Upper limit of normal
